# Standardized Brazilian green propolis extract (EPP-AF®) in COVID-19 outcomes: a randomized double-blind placebo-controlled trial

**DOI:** 10.1038/s41598-023-43764-w

**Published:** 2023-10-27

**Authors:** Marcelo Augusto Duarte Silveira, Matheus de Alencar Menezes, Sergio Pinto de Souza, Erica Batista dos Santos Galvão, Andresa Aparecida Berretta, Juliana Caldas, Maurício Brito Teixeira, Marcel Miranda Dantas Gomes, Lucas Petri Damiani, Bruno Andrade Bahiense, Julia Barros Cabral, Cicero Wandson Luiz Macedo De Oliveira, Talita Rocha Mascarenhas, Priscila Carvalho Guedes Pinheiro, Milena Souza Alves, Rodrigo Morel Vieira de Melo, Flávia Mendes Leite, Carolina Kymie Vasques Nonaka, Bruno Solano de Freitas Souza, Nathália Ursoli Baptista, Flávio Teles, Suzete Farias da Guarda, Ana Verena Almeida Mendes, Rogério da Hora Passos

**Affiliations:** 1 D’Or Institute for Research and Education (IDOR), Hospital São Rafael, Avenida São Rafael 2152, São Marcos, Salvador, BA 41253-190 Brazil; 2 Development and Innovation Department, Apis Flora Indl. Coml. Ltda, Rua Triunfo 945, Subsetor Sul 3, Ribeirão Preto, SP 14020-670 Brazil; 3 Academic Research Organization, Hospital Israelita Albert Einstein, Av. Albert Einstein, 627, Morumbi, São Paulo, SP 05652-000 Brazil; 4 School of Medicine, Federal University of Bahia, Rua Augusto Viana s/n, Canela, Salvador, BA 40110-909 Brazil; 5 Gonçalo Moniz Institute, Oswaldo Cruz Foundation (FIOCRUZ), Bahia, 40296-710 Brazil; 6 School of Medicine, Federal University of Alagoas, Av. Lourival de Melo Mota S/N, Tabuleiro do Martins, Maceió, Alagoas 57072-900 Brazil

**Keywords:** Microbiology, Diseases

## Abstract

SARS-CoV-2 and its different variants caused a “wave and wave” pandemic pattern. During the first wave we demonstrated that standardized Brazilian green propolis extract (EPP-AF®) reduces length of hospital stay in adult patients with COVID-19. Afterwards, we decided to evaluate the impact of EPP-AF in hospitalized patients during the third wave of the pandemic. BeeCovid2 was a randomized, double-blind, placebo-controlled clinical trial in hospitalized COVID-19 adult patients. Patients were allocated to receive an oral dose of 900 mg/day of EPP-AF® or placebo for 10 days. The primary outcome was length of hospital stay. Secondary outcomes included safety, secondary infection rate, duration of oxygen therapy dependency, acute kidney injury and need for intensive care. Patients were followed up for 28 days after admission. We enrolled 188 patients; 98 were assigned to the propolis group and 90 to the placebo group. The post-intervention length of hospital stay was of 6.5 ± 6.0 days in the propolis group versus 7.7 ± 7.1 days in the control group (95% CI − 0.74 [− 1.94 to 0.42]; *p* = 0.22). Propolis did not have significant impact on the need for oxygen supplementation or frequency of AKI. There was a significant difference in the incidence of secondary infection between groups, with 6.1% in the propolis group versus 18.9% in the control group (95% CI − 0.28 [0.1–0.76], *p* = 0.01). The use of EPP-AF was considered safe and associated with a decrease in secondary infections. The drug was not associated with a significant reduction in length of hospital stay. ClinicalTrials.gov (NCT04800224).

## Introduction

Coronavirus disease 2019 (COVID-19) caused by severe acute respiratory syndrome coronavirus 2 (SARS-CoV-2) has been a significant concern regarding its global impact on healthcare settings^1^.

After the viral replication phase, there is an enormous immunological and inflammatory challenge since both innate and adaptive immunity may be disorderly activated by SARS-CoV-2 infection^2^. The magnitude of these inflammatory responses may result in local and systemic organ damages^2^. Furthermore, immunological imbalance may be associated with worse outcomes and greater vulnerability, causing, for example, secondary infections (bacterial or fungal infection)^3,4^.

Propolis is a natural resin produced by bees from various parts of plants. It has several properties, such as antioxidant, anti-inflammatory and immunomodulatory activities^5^. Besides these properties, experimental and clinical trials show that propolis is capable of targeting viruses^6–10^. Green propolis-derived compounds may generate negative feedback in the expression of transmembrane serine protease 2 (TMPRSS2), which engages in the activation of spike protein of viruses. It also interferes with angiotensin-converting enzyme 2 (ACE2) anchoring, therefore limiting the entry of virus in a cell^7,8^. Also, there is evidence that some propolis compounds are capable of reducing the activation of p21-activated kinase-1 (PAK1), overexpressed in the lungs and an important target used by viruses to both shield themselves from adaptive immunological responses and locally stimulate an exaggerated inflammatory response^7–10^.

A recent open-label, randomized clinical trial in adults hospitalized due to COVID-19 has demonstrated the safety and efficacy of Brazilian green propolis (EPP-AF) to decrease length of hospital stay among non-vaccinated patients with moderate to severe symptoms of the disease (including those patients in mechanical ventilation and intensive care therapy) during the first wave of the pandemic^11^.

As the pandemic progressed and in light of experimental evidence and properties of propolis, additional clinical trials to gather more evidence are needed. We designed this randomized, double-blind clinical trial to assess safety and impact of EPP-AF on this population during the third wave of the pandemic.

## Methods

### Trial design and oversight

BeeCovid2 was a randomized, double-blind, placebo-controlled clinical trial to evaluate safety and efficacy of EPP-AF in hospitalized patients with COVID-19 who were not in mechanical ventilation. The study was conducted at Hospital São Rafael, a tertiary 600-bed hospital in Salvador, Bahia, northeastern Brazil, from April to August 2021. The protocol was approved by the local Ethics Committee (registration number 43265321.9.0000.0048) on February 25, 2021, and the trial was registered on March 16, 2021, on ClinicalTrials.gov (NCT04800224). More details can be found in the previously published study protocol^12^.

The study was conducted in accordance with the principles of the Declaration of Helsinki and the guidelines of Good Clinical Practice of the International Harmonization Conference. All participating patients and/or their legal representatives were duly informed about objectives and risks of participation before signing the informed consent form. The informed consent form is electronically signed, stored on the Research Electronic Data Capture (REDCap) platform, and automatically made available to participants via download. The electronic informed consent form was evaluated and approved by the local Ethics Committee.

All authors guarantee data integrity and fidelity to the study protocol. This study was an initiative of the principal investigator and correspondent. An independent commission from D’Or Institute for Education and Research, an institutional platform to support research, monitored the research documents, ensuring greater information security and fidelity. Any novel information arising from the study were promptly reported to the Ethics Committee during the study.

### Participants

It was considered eligible to study participation patients aged 18–80 years hospitalized with SARS-CoV-2 infection, diagnosis confirmed by reverse transcriptase-polymerase chain reaction (RT-PCR) and symptoms starting within 14 days of the randomization date.

Exclusion criteria included patients undergoing mechanical ventilation at randomization, pregnancy or lactation, known hypersensitivity to propolis, propolis use within the prior 30 days, active cancer, human immunodeficiency virus positivity, solid organ or bone marrow transplant, use of immunosuppressive drugs, bacterial or fungal infection at randomization, sepsis or septic shock, inability to administer medication orally or via a nasoenteral tube, known liver failure, advanced heart failure (New York Heart Association [NYHA] class III or IV) or end-stage kidney disease.

### Procedures

Trial randomization was stratified based on clinical parameters related to the need for supplemental oxygen as follows: no use of oxygen, catheter ≤ 5 L/min, nasal catheter > 5 L/min or non-rebreather mask, continuous positive airway pressure (CPAP), or high-flow nasal cannula. Randomization was carried out from permuted blocks listed on the REDCap platform. The permuted block (four patients per block) randomization sequence, including stratification, was prepared by a statistician not involved in the trial. To minimize bias, design and allocation concealment were performed by a trained professional who is not connected to the study.

Eligible patients were randomly assigned at a 1:1 ratio to receive Propomax® capsules produced with dehydrated standardized Brazilian green propolis extract, EPP-AF®, for 10 days at 900 mg/day (three 100-mg capsules, three times per day) or placebo (three capsules, three times per day). Hard gelatin capsules with magnesium stearate (1%), silicon dioxide (0.1%), and microcrystalline cellulose qsp (320 mg) were prepared as placebo formulations. Placebo packages had the same labelling of the active product. They were also opaque and had a security system to prevent improper opening. All capsules had the same organoleptic characteristics, making propolis and placebo capsules indistinguishable. The study was double-blinded, so neither the patients, the healthcare providers nor the principal investigator knew to which group the patients were allocated.

Both groups received standard-of-care treatment that could include supplemental oxygen, noninvasive or invasive ventilation, corticosteroids, antibiotics and/or antivirals, vasopressor support, renal replacement therapy, intra-aortic balloon pump (IABP), and extracorporeal membrane oxygenation (ECMO), as needed. Supportive treatment was freely selected at the sole discretion of assisting clinical teams in accordance with individual needs of patients, without any kind of interference from researchers.

The propolis extract used in this study was prepared from a single batch to guarantee uniformity. High performance liquid chromatography (HPLC) was performed in the propolis batch before the manufacturing of capsules. The standardized Brazilian green propolis extract, which is composed mainly of green propolis produced in southeastern Brazil and processed with specific extraction and drying processes, was selected for this study due to its batch-to-batch reproducibility^13,14^. A 900-mg/day dose proportionally offers 47.7 mg of total flavonoids expressed as quercetin, and 121.5 mg of total phenolics expressed as gallic acid^15,16^. The dose of standardized Brazilian green propolis extract was defined based on previous studies that used similar doses without adverse effects^11,17,18^.

Patients were evaluated daily during hospitalization, from days 1 to 28. Patients who were discharged in less than 10 days have completed treatment at home and were followed up by telephone as a means to obtain relevant safety data on the study medication.

### Outcomes

The primary endpoint was the time to clinical improvement, defined as the difference in days in length of hospital stay between groups.

The secondary outcomes included percentage of participants who needed mechanical ventilation (MV), secondary infection (defined as cultures of blood, urine or tracheal aspirate positive for fungi or bacteria), incidence of acute kidney injury, as well as need for renal replacement therapy, vasoactive drugs, IABP, or ECMO. We have also assessed the mortality rate by 28 days.

Acute kidney injury (AKI) was defined according to the Kidney Disease: Improving Global Outcomes (KDIGO) as stage 1 (increase in serum creatinine of 0.3 mg/dl in 48 h or increase in baseline serum creatinine by 1.5 to 1.9 times in 7 days), stage 2 (increase in serum creatinine by 2.9 times in 7 days), or stage 3 (threefold or more increase in serum creatinine in 7 days or initiation of renal replacement therapy).

### Safety

Patients were monitored to ensure safety, which is the major premise of the entire study. Adverse events (AE) that may compromise safety or were regarded as serious or severe (pregnancy, life-threatening illness, new hospitalization, or death) were reported to the local research ethics committee (within 24 h). Adverse events were classified according to the National Cancer Institute Common Terminology Criteria for Adverse Events, version 4.03.

### Statistical analysis

The mean length of hospital stay in the control group of BeeCovid study was 12.6 days with a standard deviation of 6.5 days^11^. Based on this premise, a study with 200 individuals allocated 1:1 has at least a 90% ability to identify an average effect for 3 days of hospital stay between propolis and placebo groups.

The main analysis of the study was conducted under the intention-to-treat principle. The modified intention-to-treat population consisted of all patients who were randomized and received either EPP-AF or placebo.

The primary outcome of the study was defined as length of hospital stay from randomization to 28 days if the patient remained hospitalized after that period. Additionally, if the patient did not survive hospitalization, length of hospital stay was considered as equal to 28 days, even when death occurred less than 28 days after randomization. Data from patients who could not be reached to complete the 28-day follow-up were censored at hospital discharge. The between-group comparison was evaluated by generalized additive models assuming beta-binomial distribution, with adjustments by stratification variable (baseline type of oxygenation) and had 95% confidence intervals.

Secondary outcomes, such as the need for dialysis and mortality, were evaluated by logistic regression models and presented relative to chance with respective 95% confidence intervals. Continuous outcomes were described by means and standard deviations and compared by generalized regression models with distributions that best fit the data. More details are provided in the study protocol^12^. All secondary outcomes were adjusted by the stratification variable. Unadjusted models were submitted to sensitivity analyses. Statistical analyses were performed using R software (R Core Team, Vienna, Austria).

## Results

### Participant characteristics

**Figure 1:**
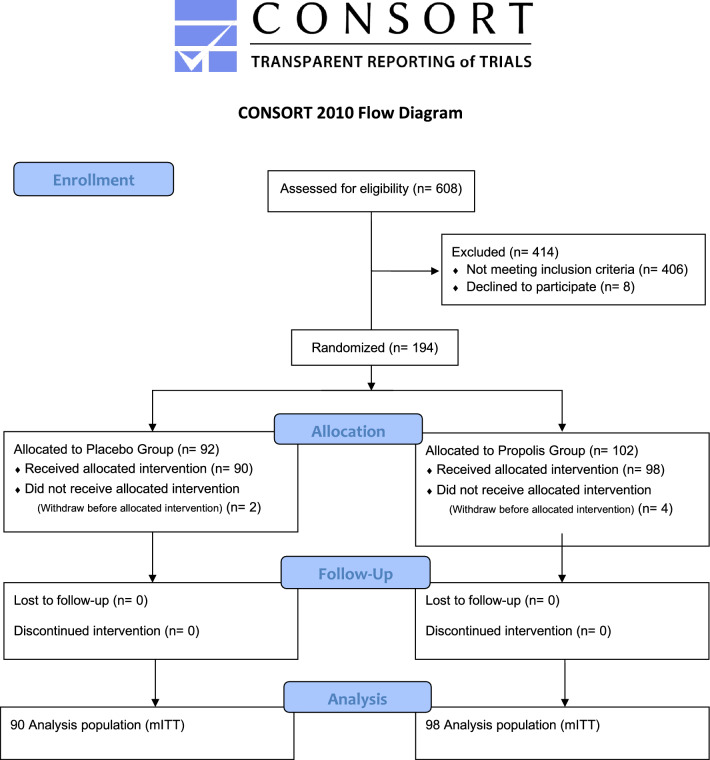
Consort flow diagram for the BeeCovid2 study.

Of the 608 patients who were assessed for eligibility, 194 met inclusion criteria. All of them were enrolled and randomized before initiating treatment: 92 patients were assigned to control group and 102 to intervention group (EPP-AF). Six patients were excluded after randomization and before receiving the medication. There were eight more participants in the intervention group in the randomization with four participants per block. However, this has not interfered with the statistical analysis. Reasons for exclusion are described in Fig. 1. The remaining patients were followed up and all of them were included in the final analysis.

The mean (± standard deviation) age of patients in this trial was 47.3 ± 12.4 and 62.8% were men (Table 1). Overall, 21.9% of patients were vaccinated for COVID 19 and 25.5% had hypertension, 12.8% were obese, 9.6% had diabetes, and only 3.2% had chronic pulmonary obstructive disease. The median (interquartile range) time from symptom onset to randomization was 10 days (8.0; 11.0] in placebo group and 9 days [8.0; 11] in EPP-AF group. Demographic and clinical characteristics of the study population are shown in Table 1.

**Table 1:** Baseline characteristics of patients of mITT^a^ population.

Characteristics	Propolis (n = 98)	Placebo (n = 90)	Total (n = 188)
Sex, Male	54/98 (55.1%)	64/90 (71.1%)	118/188 (62.8%)
Age, years, mean ± SD	47.9 ± 12.5	46.7 ± 12.4	47.3 ± 12.4
Vaccinated for COVID 19	20/98 (20.4%)	21/90 (23.3%)	41/187 (21.9%)
Clinical presentation			
Oxygen support			
None	66/98 (67.3%)	58/90 (64.4%)	124/188 (66.0%)
O2 catheter ˂ 5L/min	27/98 (27.6%)	26/90 (28.9%)	53/188 (28.2%)
O2 catheter ≥ 5L/min, high-flow or NIV	5/98 (5.1%)	6/90 (6.7%)	11/188 (5.9%)
Tomography results			
Clean image	3/97 (3.1%)	1/90 (1.1%)	4/187 (2.1%)
< 25%	12/97 (12.4%)	11/90 (12.2%)	23/187 (12.3%)
25–50%	63/97 (64.9%)	62/90 (68.9%)	125/187 (66.8%)
50–75%	19/97 (19.6%)	16/90 (17.8%)	35/187 (18.7%)
Temperature, ºC	36.5 ± 1.1	36.5 ± 1.1	36.5 ± 1.1
SpO2 on admission < 93%	12/97 (12.4%)	10/89 (11.2%)	22/186 (11.8%)
RR on admission > 24 irpm	4/97 (4.1%)	6/90 (6.7%)	10/187 (5.3%)
SBP < 90 mmHg	2/94 (2.1%)	0/83 (0.0%)	2/177 (1.1%)
Randomization in ICU	26/94 (27.7%)	25/83 (30.1%)	51/177 (28.8%)
Comorbidities			
Diabetes	8/98 (8.2%)	10/90 (11.1%)	18/188 (9.6%)
Hypertension	26/98 (26.5%)	22/90 (24.4%)	48/188 (25.5%)
COPD	1/98 (1.0%)	5/90 (5.6%)	6/188 (3.2%)
Obesity	12/98 (12.2%)	12/90 (13.3%)	24/188 (12.8%)
Stroke	0/98 (0.0%)	0/90 (0.0%)	0/188 (0.0%)
Myocardial infarction	1/98 (1.0%)	0/90 (0.0%)	1/188 (0.5%)
Baseline treatment			
Antibiotics	63/97 (64.9%)	63/90 (70.0%)	126/187 (67.4%)
Tamiflu	0/97 (0.0%)	1/90 (1.1%)	1/187 (0.5%)
Hydroxychloroquine	3/97 (3.1%)	2/90 (2.2%)	5/187 (2.7%)
Corticosteroids	82/97 (84.5%)	83/90 (92.2%)	165/187 (88.2%)
Tocilizumab	2/97 (2.1%)	5/89 (5.6%)	7/186 (3.8%)
Pentaglobin	1/97 (1.0%)	1/90 (1.1%)	2/187 (1.1%)
Remdesivir	0/61 (0.0%)	2/47 (4.3%)	2/108 (1.9%)

COPD, chronic obstructive pulmonary disease; ICU, intensive care unit; NIV, non-invasive ventilation; O2, oxygen; RR, respiratory frequency; SBP, systolic blood pressure; SD, standard deviation; SpO2, peripheral O2 saturation.

^a^mITT: Modified intention-to-treat population (all randomized patients < 80 years who received propolis or placebo at least one time).

At randomization, most patients have not received oxygen support (66.0%), 28.2% were using low-flow catheter (< 5 l/min) and 5.9%, non-invasive mechanical ventilation or high-flow catheter, with no difference between groups (Table 1). Fifty-one patients (28.8%) were being treated in intensive care unit. Follow-up information at day 28 after admission for the primary outcome was collected for all 188 patients. The use of antibiotics, Tamiflu, hydroxychloroquine, corticosteroids, tocilizumab, Pentaglobin and remdesivir were similar among all groups (Table 1).

### Outcomes

**Figure 2:**
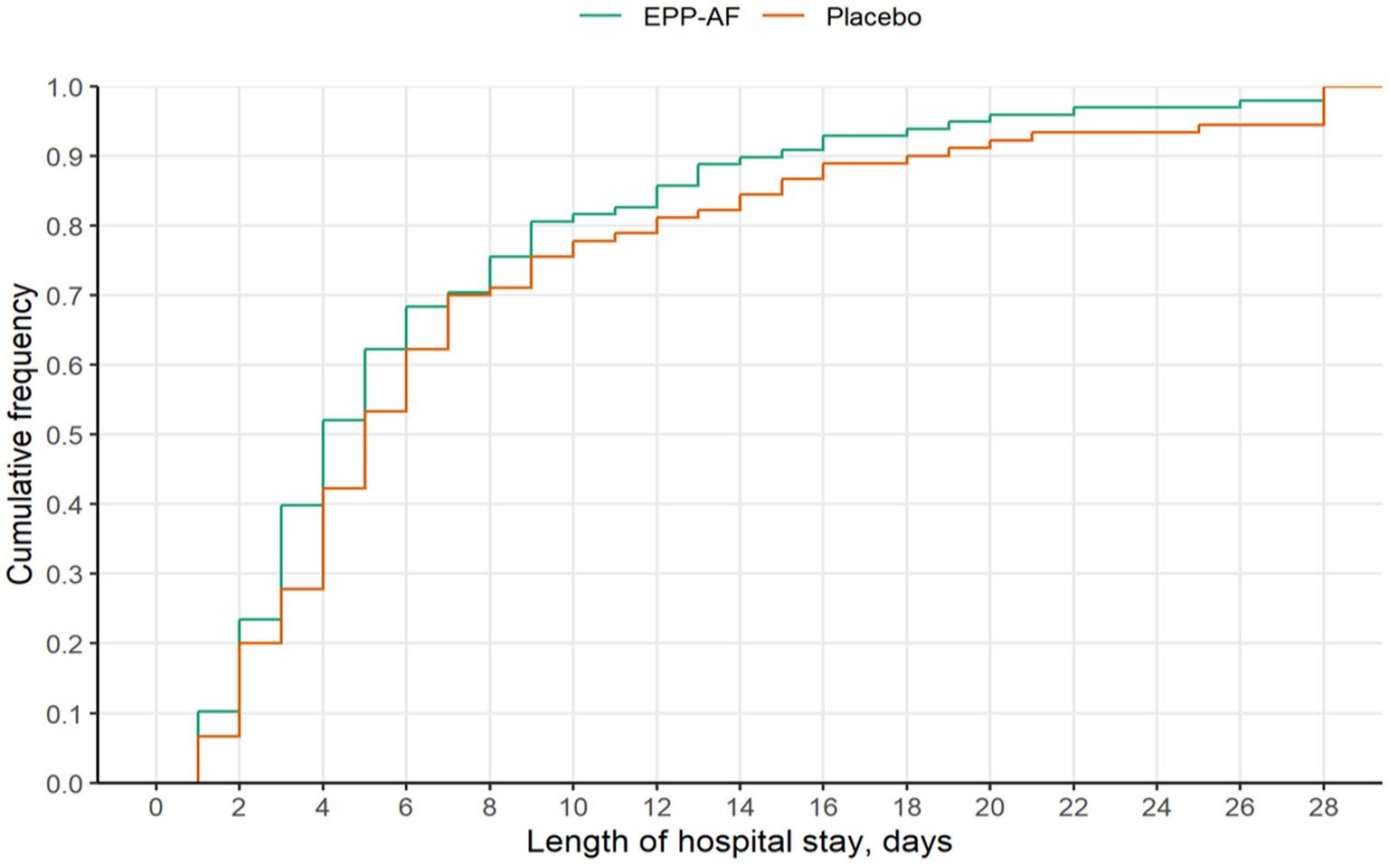
Cumulative frequencies of COVID-19 patient primary outcomes. The length of hospital stay after randomization for propolis group and placebo group.

The primary outcome*,* length of hospital stay at 28 days, was similar between both groups. The propolis group had 6.5 ± 6.0 days versus 7.7 ± 7.1 days in placebo group (95% CI – 0.74 [− 1.94 to 0.42]; *p* = 0.22) (Table 2, Fig. 2).

**Table 2:** BeeCovid Study Outcomes (mITT^c^ population).

Outcomes	EPP-AF	Placebo	Statistical effect	Adjusted^a^		Unadjusted	
	(n = 98)	(n = 90)		Estimate (95% CI)	*p*-value	Estimate (95% CI)	*p*-value
Primary outcome							
Length of hospital stay, days							
Mean (95% CI)	6.48 ± 5.99 (n = 98)	7.72 ± 7.06 (n = 90)	MD	− 0.74 [− 1.94 to 0.42]	0.22	− 1.14 [− 3.16 to 0.82]	0.26
Median (IQR)	4 [3-8]	5 [3-9]	–	–	–	–	–
Length of hospital stay (vaccination status), days							
Vaccinated	8.2 ± 6.8 (n = 20)	8.7 ± 8 (n = 21)	MD	0.57 [− 3.43 to 4.58	0.78	− 0.47 [− 4.48 to 3.55]	0.82
Non-vaccinated	6.0 ± 5.7 (n = 78)	7.4 ± 6.8 (n = 69)	MD	− 1.56 [− 3.64 to 0.53]	0.14	− 1.40 [− 3.52 to 0.73]	0.20
Secondary outcomes							
Secondary infection	6/98 (6.1%)	17/90 (18.9%)	OR	0.28 [0.1 to 0.76]	0.01	0.28 [0.11 to 0.75]	0.01
Acute kidney injury, No. (%)	13/98 (13.3%)	16/90 (17.8%)	OR	0.71 [0.32 to 1.57]	0.40	0.71 [0.32 to 1.57]	0.39
AKI KDIGO							
1	11/98 (11.2%)	13/90 (14.4%)					
2	1/98 (1%)	3/90 (3.3%)					
3	1/98 (1%)	0/90 (0%)					
Renal Replacement Therapy, No. (%)	0/98 (0%)	0/90 (0%)	OR	–	–	–	1.00
Need of vasopressor use, No. (%)	5/97 (5.2%)	5/90 (5.6%)	OR	0.98 [0.27—3.58]	0.97	0.92 [0.26 to 3.30]	0.90
Need of MV, No. (%)	10/98 (10.2%)	12/90 (13.3%)	OR	0.75 [0.31 to 1.85]	0.54	0.74 [0.30 to 1.80]	0.51
Days in MV, mean (SD)	0.71 ± 2.4 (n = 98)	0.86 ± 2.77 (n = 90)	MD	− 0.05 [− 0.78 to 0.59]	0.84	− 0.15 [− 0.99 to 0.64]	0.70
ICU admission, No. (%)	23/97 (23.7%)	24/89 (27%)	OR	0.85 [0.44 to 1.66]	0.63	0.84 [0.43 to 1.63]	0.61
Need for IAB, No. (%)	0/98 (0%)	0/90 (0%)	OR	–	–	–	1.00^b^
Need for ECMO, No. (%)	0/98 (0%)	0/89 (0%)	OR	–	–	–	1.00^b^
Death, No. (%)	0/98 (0%)	0/90 (0%)	OR	–	–	–	1.00^b^
Exploratory outcomes, No. (%)							
Thromboembolic events	2/98 (2%)	1/90 (1.1%)	OR	2.17 [0.18 to 25.59]	0.54	1.85 [0.17 to 20.8]	0.62
Bleeding	1/98 (1%)	1/89 (1.1%)	OR	0.96 [0.06 to 16.22]	0.98	0.91 [0.06 to 14.72]	0.95
Platelets < 100.000	4/98 (4.1%)	5/90 (5.6%)	OR	0.70 [0.18 to 2.72]	0.61	0.72 [0.19 to 2.78]	0.64
Neurological alterations	1/98 (1%)	0/90 (0%)	OR	–	–	–	1.00^b^
Neurological lesions	0/98 (0%)	0/90 (0%)	OR	–	–	–	1.00^b^
Leukocytes < 1000	1/98 (1%)	0/90 (0%)	OR	–	–	–	1.00^b^

ECMO, extracorporeal oxygenation membrane; IAB, intra-aortic balloon; ICU, intensive care unit; MD, mean differences; MV, mechanical ventilation; OR, odds ratio; POR, proportional odds ratio; SD, standard deviation.

^a^All models were adjusted for oxygen support at baseline (stratification variable): None; O^2^ catheter < 5L; O^2^ catheter ≥ 5L, or high flow, or non-invasive ventilation.

^b^Fisher’s exact test.

^c^mITT: Modified intention-to-treat population (all randomized patients < 80 years who received propolis or placebo at least one time).

There was a significant difference in incidence of secondary infection between groups − 6.1% in propolis group versus 18.9% in placebo group (95% CI − 0.28 [0.1–0.76], *p* = 0.01). There were no significant differences in any of the other secondary outcomes.

The incidence of AKI was similar between groups: 13.3% and 17.8% in propolis and placebo groups, respectively (95% CI − 0.71 [0.32–1.57]; *p* = 0.40). No patient needed renal replacement therapy. Only five patients in each group required vasopressor drugs (95% CI − 0.98 [0.27–3.58] *p* = 0.97) (Table 2).

The percentage of patients who were admitted to intensive care unit (ICU) was 23.7% in propolis group and 27% in placebo group (95% CI − 0.85 [0.44–1.66]; *p* = 0.63). The use of mechanical ventilation was low in both groups, with no difference between them: 10.2% in propolis group and 13.3% in placebo group (95% CI − 0.75 [0.31–1.85]; *p* = 0.54). Furthermore, no patient of this trial required dispositive devices for hemodynamic support, such as IABP and ECMO. Also, no deaths were registered in both groups.

A small sample of patients was submitted to dosage of interleukins at baseline (at randomization, before receiving the study intervention) and after five days (Supplementary Table S1).

### Adverse events

Adherence to the trial intervention did not differ according to the treatment group. No patient discontinued propolis treatment due to side effects.

The most severe adverse event overall was shock/need for vasoactive drugs, which occurred in 5.6% of patients in standard-of-care group versus 5.2% in propolis group (*p* = 0.97) The second most common adverse event was acute respiratory failure resulting in need of mechanical ventilation, which occurred at a rate of 13.3% in standard-of-care group and 10.2% in propolis group (Table 2).

There was a slightly higher, but statistically irrelevant (*p* = 0.085) number of patients with at least one reported AE in intervention group: 9 (9.2%) vs. 2 patients (2.2%). Gastrointestinal adverse events occurred in five patients in propolis group, especially epigastric pain, gastroesophageal reflux, and nausea, while only one patient in control group reported such adverse events.

Headache was the sole neurological event reported and it occurred in only one patient in intervention group. Only one patient in control group had pollakiuria. Episodes of itching and rash were observed in three patients of propolis group (Table 3). Overall, the proportion of patients experiencing AEs was similar in both groups.

**Table 3:** Reported adverse events in mITT^b^ population during follow-up.

Adverse events (AE)	EPP-AF (n = 98)	Placebo (n = 90)	*p* value^a^
Patients with at least one reported AE	9/98 (9.2%)	2/90 (2.2%)	0.085
Headache	1/98 (1.0%)	0/90 (0%)	1
Nausea	2/98 (2.0%)	0/90 (0%)	0.515
Rash	1/98 (1.0%)	0/90 (0%)	1
Itching	2/98 (2.0%)	0/90 (0%)	0.515
Epigastralgia	2/98 (2.0%)	1/90 (1.1%)	1
Pollakiuria	0/98 (0%)	1/90 (1.1%)	1
Gastroesophageal reflux	1/98 (1.0%)	0/90 (0%)	1

^a^Fisher’s exact test.

^b^mITT: Modified intention-to-treat population (all randomized patients < 80 years who received propolis or placebo at least one time).

## Discussion

In this randomized, double-blind, placebo-controlled study, the use of standardized Brazilian green propolis extract associated with standard-of-care treatment reduced the length of hospital stay by one day compared to a placebo group among hospitalized adult patients with mild to moderate COVID-19. This trend was more noticeable among non-vaccinated patients despite no statistical significance. On the other hand, the use of propolis was associated with three times fewer secondary infections.

A recent analysis in a tertiary hospital has demonstrated that each day of hospitalization costs around US$ 900^19^. Hospital costs arising from COVID-19 infections are a great concern around the world. The ability to reduce hospital stay in one day, in addition to a significant reduction in the rate of secondary infection, has a significant economic impact. An economic analysis was not the subject of the present study, but these results certainly have a positive effect from a public health point of view.

The first randomized study to use EPP-AF in moderate to severe COVID-19 non-vaccinated patients showed a significant decrease in length of hospital stay^11^. However, the trial was conducted during the first wave of the disease when hospital stays were longer and there were no patients vaccinated. Also, patients using orotracheal intubation at randomization were not excluded in this previous study^11^. In the present study, almost one quarter of the patients were vaccinated. Furthermore, between the first and third COVID-19 waves, progress was made regarding drug therapies, and scientists have gained more knowledge about the disease. Also, the virulence of SARS-COV-2 has substantially changed. This new scenario justifies the difference in length of hospital stay between studies, suggesting that the benefits of propolis adjunct therapy are greater in cases with poorer prognosis, as in the first study^11^. Albeit no statistical significance, the difference in length of hospital stay in the present study may show a trend when combined with findings of the first trial.

SARS-CoV-2 infection is more associated with secondary infections when compared to non-infected patients^20^. Coronavirus infections cause an unprecedented immunological imbalance and make the body systems more vulnerable^3,21^. Additionally, the combination of viral infection and use of immunosuppression-inducing drugs increases the risk of secondary infections, such as bacterial and fungi ones^22,23^. According to a recently published systematic review, 7% to 51% of critically ill COVID-19 patients may have a secondary infection, and it contributes to a higher mortality^24^. Another recent observational study has demonstrated that secondary pulmonary infection is the most common issue (65%), followed by bloodstream infection of uncertain origin and infections related to the central venous catheter^22^. One of the most interesting findings of this study was the reduction of secondary infections (by bacteria and fungi, threefold decrease when compared with placebo group), defined as positive cultures (of specimens such as blood, urine or tracheal aspirate). We know that the occurrence of secondary infections among COVID-19 patients is frequent and may negatively impact the evolution of patients, as well as substantially increase hospitalization costs and mortality rates^25^. Propolis promotes immunomodulation and is not associated with immunodepression^5,6^. It also has antimicrobial activities against viruses, fungi, and bacteria. Despite its inherent characteristics, it is not possible to ensure that this finding has any relationship with this mechanism. More studies are needed to confirm this finding.

BeeCovid2 has demonstrated that the use of standardized Brazilian green propolis extract in the dose used is safe, even among critically ill patients, without a single case of hospitalization or readmission to the hospital due to EPP-AF side effects. The safety analysis was performed throughout the entire study period. Previous randomized studies that used EPP-AF had already shown the safety profile of this medication, even in patients with chronic kidney disease undergoing hemodialysis^11,17,26^. Although shock and MV were on the list of severe adverse events, we cannot link the occurrence of such events to the drug tested due to the severity of the disease or even secondary infections in every patient who had them, especially because it has happened in both groups tested.

Acute kidney injury may occur in a significant number of COVID-19 patients^27^. The mechanism of acute kidney injury is multifactorial, involving endothelial dysfunction with coagulative dysfunction, organ crosstalk, drug nephrotoxicity, cytokine release syndrome (a complex process driven by virus-mediated injury), rhabdomyolysis, and renin–angiotensin–aldosterone system impairment^27,28^. It is known that some medications already tested in patients with COVID-19 may be associated with an increased risk of acute kidney injury^29^. In the first trial, a lower AKI rate was identified in the group receiving a higher dose of EPP-AF^11^. The data were consistent with an experimental study in model of sepsis showing that the use of EPP-AF is associated with lower stimulation of TRL4 renal expression and lower activation of NFK-B system, as well as lower interleukin infiltration in renal tissue^30^.

Our trial has several limitations. Due to the lack of supplies during the pandemic, glomerular filtration was assessed using creatinine levels calculated by dry chemistry method, which suffers interference by dipyrone (metamizole), drug widely administered to fever and pain control^31^. Other limitations of the study included: study conducted in a single center and phone monitoring of patients discharged from hospital until the end of EPP-AF treatment period (10 days). Data concerning potential benefits on long Covid syndrome were not measured. Therefore, it is not possible to infer medium- or long-term effects. On the other hand, the strengths of the trial are its methods (randomized, double-blind, placebo-controlled), lack of access by the research team to any therapeutic clinical decision, as well as the performance of a safety test of the drug among critically ill patients with COVID-19 in a new phase of the pandemic, with new drugs involved.

Experimental data show the potential actions of propolis against viral targets such as TMPRSS2, ACE2 receptor and PAK1, which justifies the experimental use of EPP-AF and results in new perspectives to treat diseases that lead to immuno-inflammatory dysregulation^6,11^.

We therefore conclude that coronavirus infection has a negative impact on people worldwide. This immunological challenge opens a window of opportunity to use propolis, a natural immunomodulator. This is important because propolis may be considered and tested to treat other diseases that cause immunological dysregulations. The present study provides clinical evidence that can be explored by other researchers. In this study, the EPP-AF dose tested was safe to use in adult patients with mild to moderate Covid-19. The use of the medication was not significantly associated with decreases in length of hospital stay. More studies are needed.

### Supplementary Information

Supplementary Table S1.

## Data Availability

The datasets generated and/or analyzed during the current study are available from the corresponding author upon reasonable request. The authors confirm that the data supporting the findings of this study are available within the article and its supplementary material.
