# Supplementary material for: Standardized Brazilian green propolis extract (EPP-AF®) in COVID-19 outcomes: a randomized double-blind placebo-controlled trial
Source: Sci Rep. 2023 Oct 27;13:18405. doi: 10.1038/s41598-023-43764-w (PMC10611696; doi:10.1038/s41598-023-43764-w)
Supplement: Supplementary file 1 — Supplementary Table S1. [file 41598_2023_43764_MOESM1_ESM.pdf]

Baseline comparisons	Statistic	EPP-AF	Placebo	Total	p-Value*
IL6	Median [quartiles], n	26.0 [19.5; 27.0] (n = 7)	34.0 [20.0; 48.5] (n = 11)	26.0 [19.2; 38.8] (n = 18)	0.441
	Mean ± SD	25.4 ± 8.4	43.0 ± 36.3	36.2 ± 29.6	
TNF alpha	Median [quartiles], n	7.0 [6.0; 8.0] (n = 7)	8.5 [8.0; 9.8] (n = 10)	8.0 [7.0; 9.0] (n = 17)	0.021
	Mean ± SD	7.0 ± 1.0	8.6 ± 1.2	7.9 ± 1.3	
IFN gamma	Median [quartiles], n	14.0 [11.0; 23.0] (n = 7)	9.0 [8.0; 13.0] (n = 11)	10.0 [8.0; 16.0] (n = 18)	0.219
	Mean ± SD	16.9 ± 9.5	13.0 ± 9.4	14.5 ± 9.3	

*Mann-Whitney test

Post comparisons	Statistic	EPP-AF	Placebo	Total	p-Value¹	Adjusted² model p-value
IL6	Median [quartiles], n	21.0 [15.5; 25.5] (n = 7)	22.0 [15.0; 47.5] (n = 11)	21.0 [14.5; 30.8] (n = 18)	0.467	0.331
	Mean ± SD	21.9 ± 9.7	44.7 ± 53.4	35.8 ± 42.9		
TNF alpha	Median [quartiles], n	7.0 [7.0; 7.5] (n = 7)	7.5 [7.0; 9.0] (n = 10)	7.0 [7.0; 9.0] (n = 17)	0.647	0.343
	Mean ± SD	7.4 ± 1.3	7.8 ± 1.4	7.6 ± 1.3		
IFN gamma	Median [quartiles], n	10.0 [9.0; 13.0] (n = 7)	11.0 [7.5; 18.5] (n = 11)"	10.5 [8.0; 16.2] (n = 18)	0.865	0.617
	Mean ± SD	11.4 ± 4.6	14.5 ± 9.4	"13.3 ± 7.9"		

(1) Mann-Whitney test

(2) Linear regression models adjusted for baseline values

Table S1.

A small sample of patients was submitted to dosage of interleukins during baseline period (at randomization, before receiving the study intervention) and after five days.

There was no statistical difference among groups.
